# Supplementary figures and images for: Interaction of the causal agent of apricot bud gall Acalitus phloeocoptes (Nalepa) with apricot: Implications in infested tissues
Source: PLoS One. 2021 Sep 2;16(9):e0250678. doi: 10.1371/journal.pone.0250678 (PMC8412328; doi:10.1371/journal.pone.0250678)

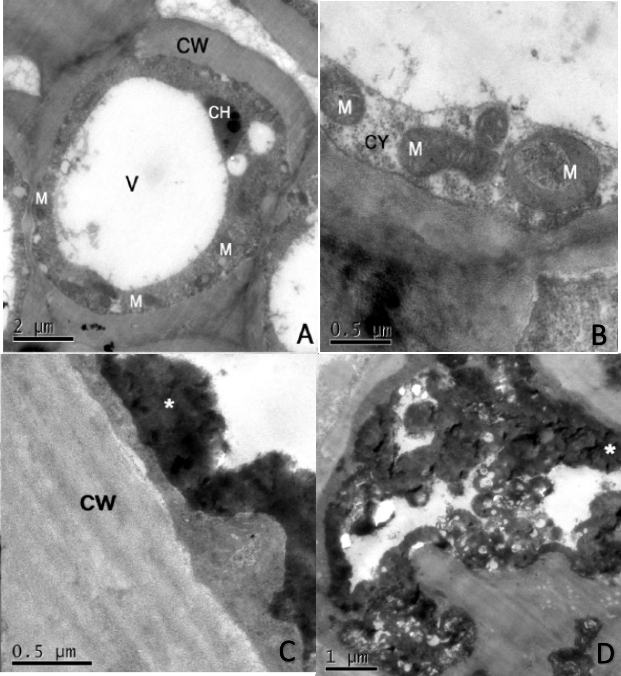

Supplement: S1 Fig — (A) Early stage of injury: the cell wall of young leaves was obviously thickened, a small amount of starch granules were formed, and the cell fluid in vacuole was clear and bright. Mid stage of injury: (B) Mitochondria are obviously expanded and irregular in shape (C) on the inner side of the cell wall there is a thin layer of electron dense material deposition, which is closely connected with the inner side of the cell wall. (D) Late stage of injury: The organelles in the cells disintegrated, and the substances with high electron density scattered in the cells. CH: chloroplast; CY: cytoplasm; CW: cell wall; M: mitochondrion; N: nucleus; S: starch; V: vacuole. Ve: vesicle; *:electron-dense granules. (JPG) [file pone.0250678.s001.jpg]
