# Supplementary material for: Interaction of the causal agent of apricot bud gall Acalitus phloeocoptes (Nalepa) with apricot: Implications in infested tissues
Source: PLoS One. 2021 Sep 2;16(9):e0250678. doi: 10.1371/journal.pone.0250678 (PMC8412328; doi:10.1371/journal.pone.0250678)
Supplement: S1 Table — (DOCX) [file pone.0250678.s003.docx]

**S1 Table**. Grading standard of apricot bud galls

| **Disease grade** | **Quantitative value** | **Grading standard** | |
| --- | --- | --- | --- |
|  |  | **Diameter of galls (cm)** | **Number of galls** |
| I | 0 | No galls on the branches of  1 to 2 years old | — |
| II | 1 | ≥ 1, ＜2 | ≤ 5 |
| III | 2 | ≥ 2, ＜2.5 | 5-10 |
| IV | 3 | ≥2.5, ＜3 (or ≥ 1＜2 ) | 6-10( or 10-15) |
| V | 4 | ≥ 3 (or ≥1, ≤2.5 ) | ＞11 (or＞15) |
