# Supplementary material for: Interaction of the causal agent of apricot bud gall Acalitus phloeocoptes (Nalepa) with apricot: Implications in infested tissues
Source: PLoS One. 2021 Sep 2;16(9):e0250678. doi: 10.1371/journal.pone.0250678 (PMC8412328; doi:10.1371/journal.pone.0250678)
Supplement: S2 Table — (DOCX) [file pone.0250678.s004.docx]

**S2 Table.** Differences between infested bud and healthy bud

| **Parameters (μm)** | **Infested bud** | **Healthy bud** |
| --- | --- | --- |
| Width of lower part axis | 1500.8±65.6 A | 332.0±50.0 B |
| Width of upper part axis | 2838.7±67.4 A | 114.8±6.0 B |
| Epidermal thickness of immature leaves | 41.9±11.4 A | 8.3±4.6 B |
| Total thickness of immature leaves | 204.1±54.3 A | 140.3±30.9 A |
| Layer number of immature leaves | 24±5 A | 12±3 B |

Means in columns followed by different letters indicate significant difference (p < 0.01).
