# Supplementary material for: Interaction of the causal agent of apricot bud gall Acalitus phloeocoptes (Nalepa) with apricot: Implications in infested tissues
Source: PLoS One. 2021 Sep 2;16(9):e0250678. doi: 10.1371/journal.pone.0250678 (PMC8412328; doi:10.1371/journal.pone.0250678)
Supplement: S3 Table — Measured from various overwintering places in in three consecutive years in Lanzhou City, Gansu Province, China. (DOCX) [file pone.0250678.s005.docx]

##### S3 Table. Number of *Acalitus phloeocoptes* (Nalepa) in different stages. Measured from various overwintering places in in three consecutive years in Lanzhou City, Gansu Province, China

| **Overwintering places** | **1st year** | | | **2nd year** | | | **3rd year** | | | **Percent (%)** | | |
| --- | --- | --- | --- | --- | --- | --- | --- | --- | --- | --- | --- | --- |
|  | **Adult** | **Nymph** | **Egg** | **Adult** | **Nymph** | **Egg** | **Adult** | **Nymph** | **Egg** | **Adult** | **Nymph** | **Egg** |
| Gall | 39 | 8 | 6 | 33 | 10 | 9 | 40 | 6 | 5 | 70.4 | 15.1 | 12.6 |
| Branch | 1 | 0 | 0 | 0 | 1 | 0 | 0 | 0 | 0 | 0.6 | 0.6 | - |
| Bark crack | 1 | 0 | 0 | 0 | 0 | 0 | 0 | 0 | 0 | 0.6 | - | - |
| Soil | 0 | 0 | 0 | 0 | 0 | 0 | 0 | 0 | 0 | - | - | - |

Number of *Acalitus phloeocoptes* (Nalepa) of a given stage represents the average across the 3 years of study.
